# Analysis and Characterization of Optimized Dual-Frequency Vibration Energy Harvesters for Low-Power Industrial Applications

**DOI:** 10.3390/mi13071078

**Published:** 2022-07-07

**Authors:** Sofiane Bouhedma, Siyang Hu, Arwed Schütz, Fred Lange, Tamara Bechtold, Mohammed Ouali, Dennis Hohlfeld

**Affiliations:** 1 Institute for Electronic Appliances and Circuits, Faculty of Computer Science and Electrical Engineering, University of Rostock, Albert-Einstein-Str. 2, 18059 Rostock, Germany; siyang.hu@jade-hs.de (S.H.); fred.lange@uni-rostock.de (F.L.); tamara.bechtold@jade-hs.de (T.B.); dennis.hohlfeld@uni-rostock.de (D.H.); 2 Structural Mechanics Research Laboratory, Mechanical Engineering Department, Blida I University, BP 270 Route Soumâa-BLIDA, Blida 09000, Algeria; ouali.mohammed@univ-blida.dz; 3 Department of Engineering, Jade University of Applied Sciences, Friedrich-Paffrath-Str. 101, 26389 Wilhelmshaven, Germany; arwed.schuetz@jade-hs.de

**Keywords:** piezoelectricity, vibration-based energy harvesting, coupled resonators, bandwidth broadening, multi-objective optimization, multimodal structures

## Abstract

We present a multiresonant vibration energy harvester designed for ultra-low-power applications in industrial environments together with an optimized harvester design. The proposed device features dual-frequency operation, enabling the harvesting of energy over a wider operational frequency range. It has been designed such that its harvesting bandwidth range is [50, 100] Hz, which is a typical frequency range for vibrations found in industrial applications. At an excitation level of 0.5 g, a maximum mean power output of 6 mW and 9 mW can be expected at the resonance frequencies of 63.3 and 76.4 Hz, respectively. The harvester delivers a power density of 492 µW/cm^2^. Design optimization led to improved harvester geometries yielding up to 2.6 times closer resonance frequencies, resulting in a wider harvesting bandwidth and a significantly higher power output.

## 1. Introduction

The ongoing development of ultra-low-power electronics and the successive development of circuit technology has enabled a drastic decrease in the power consumption of microelectronic systems. This has motivated the research community to focus on optimizing the performance of energy transducers, such as vibration-based energy harvesters. These energy harvesters are able to use ambient energy in the form of vibrations to power electronic devices such as sensors, microcontrollers, and wireless transceivers. This opens up the possibility to develop fully autonomous and battery-free systems for industrial measurement and monitoring applications, where accessibility is limited due to the harsh environmental conditions. The goal of our work and related research work is to demonstrate that a certain amount of power can be delivered by energy harvesting and that, thereby, the life span of the battery in use can be extended until the next recharge or replacement. This will have a significant impact by considerably reducing the waste and periodical maintenance costs required for battery replacement.

Vibration energy harvesting relies on a mechanical resonator to amplify the low vibration levels into usable deflections. Such designs are tip-loaded clamped-free cantilever-based systems [1,2,3,4,5,6], which we refer to as ‘conventional harvesters’. Such approaches suffer from the fact that efficient harvesting can be ensured only if the harvester’s resonance frequency coincides with the dominant ambient vibration frequency. A difference between these frequencies results in a drastic decrease in the power output. The vast majority of realistic ambient vibration spectra in industrial environments exhibit multiple frequencies, which vary over time as the vibration source ages or changes in temperature. The conventional energy harvesters fail in such conditions. Thus, many research groups are addressing this problem, and numerous new resonator designs and harvesting schemes with broadened active bandwidth have been proposed. Considerable efforts have been invested to develop new resonator designs featuring multimodal operation, i.e., structures with suitable mode shapes appearing at close resonance frequencies. Qi et al. [7] proposed a multiresonant structure comprising a clamped–clamped piezoelectric fiber composite generator. It integrates side-mounted cantilevers, which are tuned by added masses to resonate at individual frequencies, resulting in a wider harvesting bandwidth. Lamprecht et al. [8] presented a kinetic energy harvester providing a 500 Hz-wide harvesting bandwidth, capable of harvesting energy from a multitude of machines. Xiangyang Li et al. [9] developed and analytically investigated a generalized multimode piezoelectric energy harvester, generating multiple close peaks from low-frequency ambient vibration sources. Liu et al. [10] proposed a MEMS piezoelectric power generator exhibiting improved frequency agility and power output and consisting of a cantilever array with tuned dimensions and tip-masses. Shahruz [11] designed a mechanical band-pass filter for efficient energy harvesting from a variety of vibration sources with different peak-power frequencies. Zhou et al. [12] presented a novel piezoelectric energy harvester with a multimode dynamic magnifier consisting of a tip-loaded multimode intermediate beam referred to as a magnifier and an energy harvesting beam carrying a tip-mass. The harvester exhibits a higher energy harvesting capacity compared to conventional harvesters. Xiong and Oyadiji [13] developed a new multimodal harvester design which can generate up to four close resonance frequencies over the frequency range of 10 Hz to 100 Hz with relatively large power output compared to conventional harvesters. Kim et al. [14] experimentally investigated a new two-DOF coupled system which uses both rotational and translational displacements to be potentially used as a vibration-based energy harvesting device. Tang and Zuo [15] analytically investigated and optimized a dual-mass vibration-based transducer, where two masses are connected in series, and demonstrated the ability to maximize the energy harvesting potential of the dual-mass vibration harvester when subjected to harmonic excitation. Further multimodal resonator designs, which are able to operate resonantly at multiple frequencies, were introduced in [16,17,18]. Wu et al. [19] investigated a compact piezoelectric energy harvester comprised of one main cantilever beam and an inner secondary cantilever beam. The system harvests energy at two distinct frequencies. A novel trident-shaped piezoelectric energy harvester was proposed by Upadrashta and Yang in [20] to collect power from wideband, low-frequency, and low-amplitude ambient vibrations. Several other groups [21,22,23,24] proposed multiple concepts of multiresonant piezoelectric energy harvesting devices capable of harvesting power on a wider frequency range, using both translational and rotational degrees of freedom. In general, this bandwidth broadening strategy results in considerably large structures. Additionally, it adds complexity to the conditioning circuitry, due to the multiple voltage sources. Consequently, researchers investigated other strategies for broadening the harvesting bandwidth. Challa et al. [25] studied the use of a magnetic force to alter the overall stiffness of the energy harvesting device, which enables one to change the natural frequency of the transducer. A mathematical and a numerical model have been developed for a bistable conventional cantilever-based energy harvester; in addition, an experimental investigation was presented by Stanton et al. [26]. Xu and Li [27] investigated the capabilities of a bistable vibration-based energy harvester to scavenge energy from random vibrations and demonstrated the higher efficiency of bistable energy harvesting devices compared to the traditional monostable devices. Abdelkefi and Barsallo [28] presented a thorough investigation of a novel broadband low-frequency piezoelectric energy harvester, making use of magnetic softening and stiffening. Hoffmann et al. [29] designed a coupled structure which incorporates two cantilever-based electromagnetic energy harvesters coupled via a magnetic field and investigated the total power output of the energy harvester compared to a linear reference system. Spreemann and Manoli [30] investigated vibration-based energy conversion using electromagnetic vibration transducers and discussed different designs. Furthermore, they demonstrated its potential use in powering wireless sensor nodes. Further research studied [31,32,33] an increased harvesting bandwidth by introducing bistability into the harvesting device. Challa et al. [34] proposed a self-tunable energy harvesting device that utilized the piezoelectric technique for energy harvesting and the magnetic force tuning technique for resonance frequency tenability, where the tuning process was performed by means of an energy-efficient actuator programmed to periodically adjust the distance between magnets and tune the device resonance frequency to a desired source frequency. The same approach was further investigated by Hoffmann et al. in [35], with a smart and power-efficient self-adaptive energy harvesting system which was able to adapt its eigenfrequency to the ambient operating conditions of power units. Furthermore, numerous autonomous self-adaptive and power-efficient energy harvesting systems were introduced in [36,37,38,39]. Fu et al. [40,41] presented and analyzed a broadband rotational energy harvester which uses bistability and frequency up-conversion. Kim et al. [42] proposed a multimodal harvesting platform incorporating magnetically coupled linear harvesters for low-frequency vibrations. 

**Figure 1:**
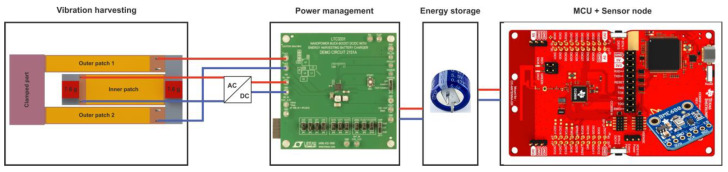
Concept of an energy-autonomous wireless sensor with Bluetooth low-energy transceiver powered by a vibration-based energy harvester.

The overall aim of our research work is to develop an optimized energy harvesting system providing higher power output and capable of delivering this power over a broader harvesting bandwidth. We propose a hybrid strategy consisting of combining multimodal operation and integration of permanent magnets for frequency tuning of harvesting modes. The latter feature supports broadening the operative bandwidth of the harvesting system. In [43,44,45], we investigated the performance and the dynamics of a compact coupled resonator design for harvesting purposes. In the present paper, we focus on investigating the operational characteristics and the optimization potential of the proposed energy harvester. Our design is a so-called ‘folded beam’ resonator design, which consists of an outer U-shaped beam mechanically connected to an inner beam. In [44,45], we proposed to use permanent magnets as masses at the free ends of the cantilevers. In addition to their inertia, they also allow for frequency tuning through external magnets. It has been demonstrated that resonance frequencies of the harvesting modes can be tuned independently. This results in extended frequency agility and superior harvesting bandwidth compared to existing approaches. The development of an energy harvester is generally application-dependent. This paper builds on our previous effort to propose a harvesting device for powering a wireless sensor node and to ensure its autonomous and battery-free operation, as summarized in Figure 1. Here, we present a thorough investigation of the proposed coupled resonator vibration energy harvesting approach. It resonates at two distinct frequencies in the range [50, 100] Hz. 

In contrast to [19], we determined the net power output of such an energy harvesting module before and after the voltage conditioning circuit. Furthermore, using a multi-objective optimization strategy, we identified design candidates with optimized performance, delivering higher power at two distinct modes appearing at closer frequencies centered around 75 Hz and resulting in an increased harvesting bandwidth. The optimization was experimentally validated. It was found that the optimized design outperformed the reference design by delivering approximately comparable power levels at the two harvesting modes appearing at closer frequencies. The proposed optimization considers the application-specific frequency pattern. Moreover, we demonstrated that the coupled resonators outperformed their equivalent decoupled designs by providing higher power output.

## 2. Modeling Approach and Characterization

This section briefly reviews the modeling approach of our dual-frequency harvester proposed in [43,44,45] as well as its extensive experimental characterization. 

### 2.1. Physical Model

**Figure 2:**
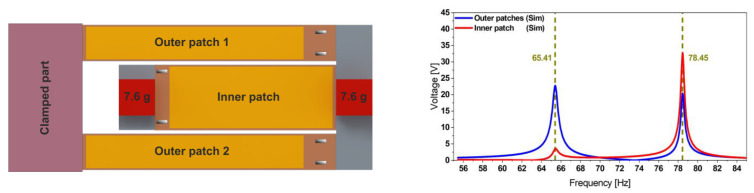
Geometry description of the reference harvester design integrating the MFC patches (**left**) together with its simulated voltage output (**right**). The transfer function illustrates the dual-frequency operation of the structure under a base acceleration amplitude of 0.5 g.

The mechanical resonator design belongs to the coupled resonators category. It consists of two identical 80 mm long beams (referred to as outer beams), mechanically coupled through a common end to a 60 mm long inner beam which extends, in turn, towards the fixed end [43]. It incorporates tip-masses of identical weight *m* = 7.6 g. The model integrates encapsulated Macro Fiber Composite^TM^ (MFC) piezoelectric patches, supplied by SMART MATERIAL Corp. (outer beams: two 60 × 7 × 0.18 mm^3^ M-8507-P2 patches; inner beam: 48 × 14 × 0.18 mm^3^ M-8514-P2 patch) [45], as depicted in Figure 2. The deformation of the piezoelectric layers causes a surface charge distribution and, consequently, a voltage across the patch electrodes. In the previous works [43,44,45], we validated our FE model results, represented in Figure 2, through a comparison of the simulation with the characterization results.

In this work, we further improve our FE model. For the sake of simplification, the piezoelectric layer of the MFC patch is considered as a piezoelectric volume with homogenous material properties instead of as a microfiber structure. The FE model integrates the piezoelectric properties of this composite material. The model also uses mode-dependent damping ratios, which are *ε*_1_ = 0.4% and *ε*_2_ = 0.2%, for the first and second mode, respectively. These values were chosen such that the amplitude at each mode matches its corresponding experimental value as described in [45].

For simplification purposes, we neglect the viscoelastic properties of the glue layer, and we consider a 58 µm thin adhesion layer with an elasticity modulus of *E* = 0.45 MPa. Realistic distributed vibration spectra excite both modes simultaneously so that individual voltage processing is mandatory.

### 2.2. Reference Harvester Characterization

This section is dedicated to a characterization of the dynamics of our reference vibration harvester design, consisting of a coupled resonator design, under different vibration modes, as well as to an estimation of its performances compared to cantilever-based harvesters.

#### 2.2.1. Mechanical Resonator

**Figure 3:**
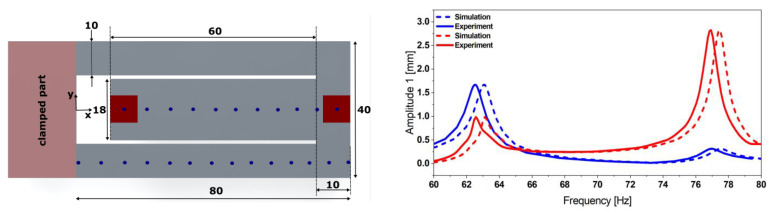
Geometry details of the coupled steel-based resonator (**left**) together with its experimental displacement transfer function at 0.5 g (**right**), measured at each free end of the structure and validating the simulations estimation provided by the corresponding FE model.

The characterization and the power output estimation of the proposed harvester design bring us back to characterizing the dynamics of the mechanical resonator. A harmonic response analysis was conducted using a sinusoidal base acceleration with an amplitude *a* = 0.5 g. The corresponding experimental values represented in Figure 3 were obtained using dedicated vibration test equipment (TV 5110 vibration test system). 

**Figure 4:**
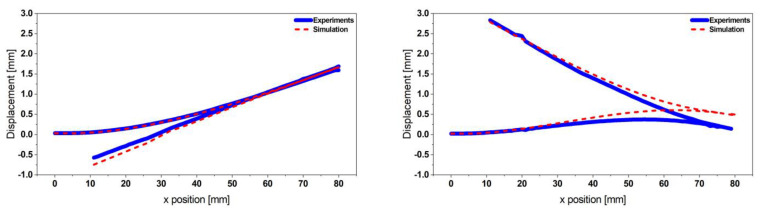
Experimental validation of the coupled resonator’s mode shapes obtained from the FE model harmonic excitation, considering a 0.5 g base acceleration (Mode 1 (**left**) and Mode 2 (**right**)).

The equipment integrates an OFV-302 interferometer sensor head from Polytech and moveable mirrors to scan the whole structure. Therefore, displacement readings enable mode shape characterization. The two fundamental mode shapes represented in Figure 4 and appearing, respectively, at the two resonance frequencies *f_1exp_* = 62.63 Hz and *f_2exp_* = 76.07 Hz match the simulation data (*f_1sim_* = 63.18 Hz and *f_2sim_* = 77.45 Hz) as represented in Figure 3.

The mode-dependent damping ratios were chosen such that the displacement amplitude of the FE model and its resonance frequencies matched the experimental data. A discrepancy of 1.2% remains for the displacement amplitude. The resonance frequencies of the FE model differ by 1.35%.

#### 2.2.2. Energy Harvester

The harvester integrates three MFC patches with the resonator considered in the above section. Two patches (M-8507-P2, 60 × 7 × 0.18 mm^3^), electrically connected in parallel, and a third patch (M-8514-P2, 48 × 14 × 0.18 mm^3^) are attached to the steel-based resonator. The material properties of the MFC patches are given in Table 1.

**Figure 5:**
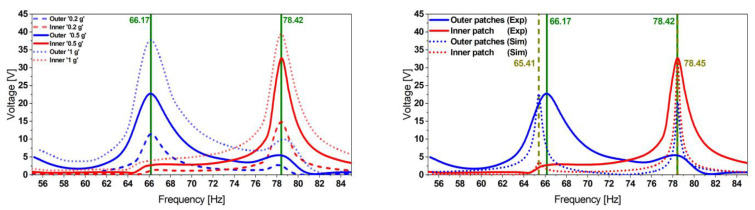
Experimental voltage transfer function of the initial reference harvester design obtained at different excitation levels (**left**) followed by an experimental validation of the FE model estimations (**right**) showing a good match in terms of frequencies as well as the voltage amplitudes.

The harvester was excited at acceleration amplitudes of 0.2, 0.5, and 1.0 g. The results presented in Figure 5 demonstrate the dual-frequency feature of the harvester. In contrast to [45], where we used an adhesive tape, here we employed epoxy-glue to attach the patches. This enhances strain transfer and yields higher voltages.

The experimentally observed resonance frequencies, *f*_1 Exp_ = 66.17 and *f*_2 Exp_ = 78.42 Hz, match the simulation results, *f*_1 Sim_ = 64.30 and *f*_2 Sim_ = 77.50 Hz. This FE model considers the adhesive layer as a material of high compliance (*E* = 450 kPa) and implements a mode-dependent damping ratio. Furthermore, the slight frequency deviation (up to 1.2%) between the model and the experimental results is caused by the MFC patch geometry simplifications and the implemented glue properties—as obtained from the supplier’s datasheet—as well as the homogeneity of the glue layer thickness. The limited reproducibility of the patch attachment and tip-masses positions constitutes an additional reason for this discrepancy.

**Figure 6:**
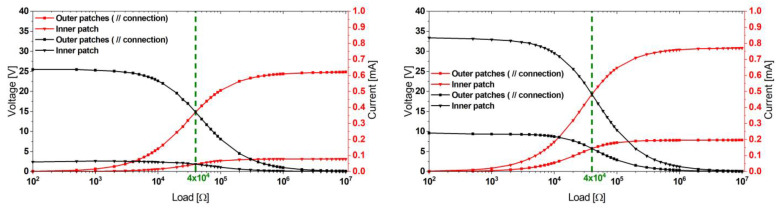
Experimental load-matching approach followed to obtain the optimum load values of the outer and inner patches of the reference design harvester at the first (**left**) and the second (**right**) modes.

Due to the capacitance of the piezoelectric patches, the power output depends on the attached load resistance. The optimum load value was experimentally determined to be 40 kΩ for the outer and inner beams, respectively, as depicted in Figure 6.

**Figure 7:**
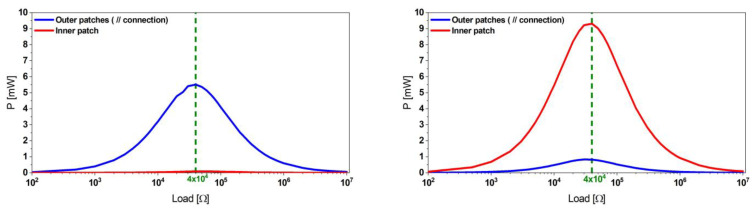
Power values of the reference harvester design at the first (**left**) and the second (**right**) mode obtained at the corresponding optimum load.

We excited the harvester at several excitation levels up to 0.5 g. The power delivery to a matched load was found to be 5.6 and 10.1 mW for the outer and inner beams, respectively, as shown in Figure 7. 

**Figure 8:**
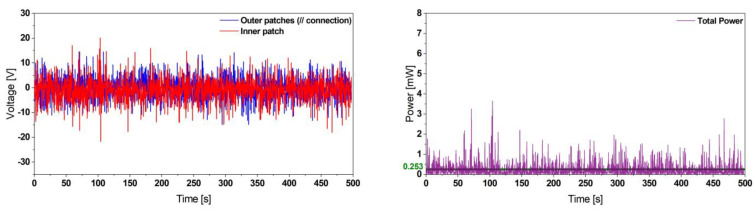
Experimental voltage (**left**) and instantaneous power output (**right**) results of the reference harvester design undergoing a noise excitation providing a power density of 0.005 g^2^/Hz. This demonstrates the harvesting capabilities of our proposed design to deliver sufficient power for ultra-low-power applications.

Furthermore, we investigated the performance of our proposed design under noise excitation. A white noise signal in the frequency range [50, 100] Hz with a power density level of 0.005 g^2^/Hz was applied. The system delivered a power of 253 µW, as represented in Figure 8.

#### 2.2.3. Power Management and Energy Storage

**Figure 9:**
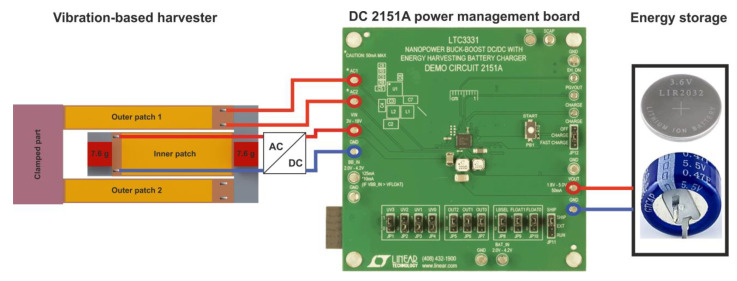
Power management and storage module used for characterization of the reference harvester design.

Piezoelectric energy harvesting bears the risk of generating voltage levels which exceed the range compatible with electronic circuits and related components, such as microcontrollers or power storage modules. Consequently, a voltage conditioning circuit is required for a reliable operation. Therefore, we evaluated the harvester’s power delivery using the power management board Analog Devices 2151A (see Figure 9), which also integrates battery charging capabilities [46]. 

The board incorporates the LTC3331 chip, which provides a regulated voltage from various energy harvesting sources. The conditioning circuitry consists of an integrated low-loss full-wave bridge rectifier and a buck converter. The rechargeable coin-cell battery powers a buck-boost converter capable of providing voltages between 1.8 and 5.0 V. Depending on the power available from the harvester and the power required by the application, the power is either supplied by the harvester or the battery. An internal prioritizer selects the suitable power source. If the harvesting source meets the power requirement needed for an autonomous operation of the system, the buck regulator is active, and the buck-boost converter is off and vice versa. The outer patches, which are electrically connected in parallel, are directly connected to the AC input of the board. Therefore, an additional bridge rectifier (FS B500-C1500 provided by DIOTEC Semiconductor AG, Heitersheim, Germany) is required, to be connected to the inner patch prior to the DC input of the power management board, since the latter provides only a single AC input.

The power management circuit efficiency, which is the ratio between the input and the output power, is summarized in Table 2. A part of the power generated by the harvester is dissipated by the electronics of the power management circuitry. We were able to estimate the net power output of our dual-frequency harvester at each mode and after the conditioning circuitry, as represented in Figure 9. 

Our overall goal is to supply sufficient power to a wireless sensor and ensure its autonomous operation in a sensor network. To do so, an energy storage module is needed. An experimental investigation demonstrated that a 470 mF super capacitor would be sufficient to run our system autonomously. Thus, we tested the capabilities of our harvester at its two respective resonance frequencies to charge the dedicated supercapacitor at an excitation level of 0.5 g. The harvester was able to charge an empty super capacitor to 99% of its nominal voltage in around 30 min.

As a summary, we demonstrated the dual-frequency feature of our harvesting device and demonstrated that our system was able to deliver enough power to a microcontroller (Texas Instrument CC-2650 Launchpad, Dallas, TX, USA) with an average power requirement of 500 µW at an average operating voltage of 2.0 V.

However, the drawback of such a design lies in the frequency spacing between the two modes, which was 10 Hz in our case. Therefore, in the following section, an optimization strategy is proposed to produce designs with better performances. 

## 3. Design Optimization and Characterization

The finite element (FE) model, described in Section 2.1, was subjected to a multi-objective optimization approach, which yields candidate designs with enhanced performances.

### 3.1. Optimization Approach

For design optimization of the folded beam piezoelectric energy harvester, we employed a two-stage global optimization strategy. In the first stage, an evolutionary algorithm (EA) samples the entire design space and identifies interesting subspaces. EA is a nature-inspired metaheuristic optimization approach. Starting from a set of initial designs, the algorithm imitates the biological process of evolution and evolves the starting population towards optimal designs via selection, crossover, and mutation. EA can be considered as a state-of-the-art optimizer, due to its robustness, wide applicability, and convenience of use. However, because of its conception, optimality cannot be guaranteed in a strict mathematical sense. Therefore, we implemented a second stage involving classical gradient-based and deterministic nonlinear programming in the subspaces identified in the first stage. For the first stage, we chose a Non-dominated Sorting Genetic Algorithm (NSGA-II), which is a state-of-the-art evolutionary algorithm. In addition to crossover and mutation, NSGA-II incorporates a mechanism called elitism, which stores a set of best designs of previous generations and makes them available in every following generation. For the second stage, Nonlinear Programming by Quadratic Lagrangian (NLPQL) was the algorithm of our choice. It is very efficient, as it approximates constraints and the derivatives of the goal function. Both algorithms are available in ANSYS^®^ Mechanical and optiSLang. The optimization of the folded beam was implemented using ANSYS^®^ Mechanical 2021R1.

**Figure 10:**
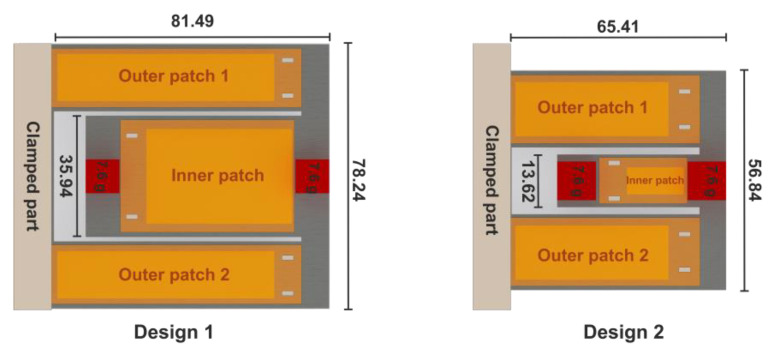
Two candidate designs with thicknesses of t=1 mm (**left**) and t=0.5 mm (**right**) obtained from the optimization approach. (Dimensions are given in mm).

**Figure 11:**
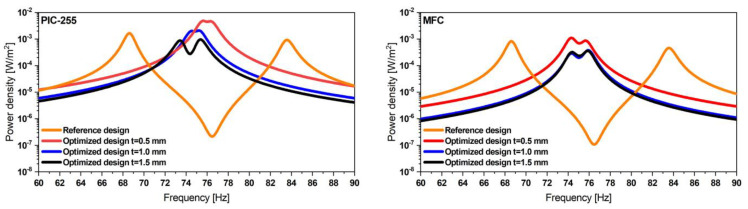
Estimated power density of the proposed optimized designs considering PIC-255 (**left**) and MFC patches (**right**), outperforming the initial reference designs in both cases. The closely spaced resonance frequencies result in an extended operative bandwidth at comparable power levels.

Due to symmetry, only a half geometry was considered. Furthermore, the power output was computed based on the mechanical stress integral, relieving the need to consider piezoelectric properties. The optimization process was first introduced in [45], where we parameterized the folded beam harvester and presented the detailed optimization process and its parameters. The optimization process was applied to a folded beam harvester with MFC patches. While design goals, parameterization, and design space remained unchanged, additional constraints were introduced, accounting for fabrication constraints, i.e., the discrete available widths of MFC patches. Figure 10 and Figure 11 as well as Table 3 present the optimized design geometry details and respective power density frequency responses for both optimization results.

The power density plots demonstrate that all optimal designs outperform the initial design significantly in terms of resonance frequency spacing. The third candidate design, considering a steel resonator with a thickness of 1.5 mm, is not shown due to its considerable size, exceeding our space limitations.

### 3.2. Optimized Harvesters Characterization

**Figure 12:**
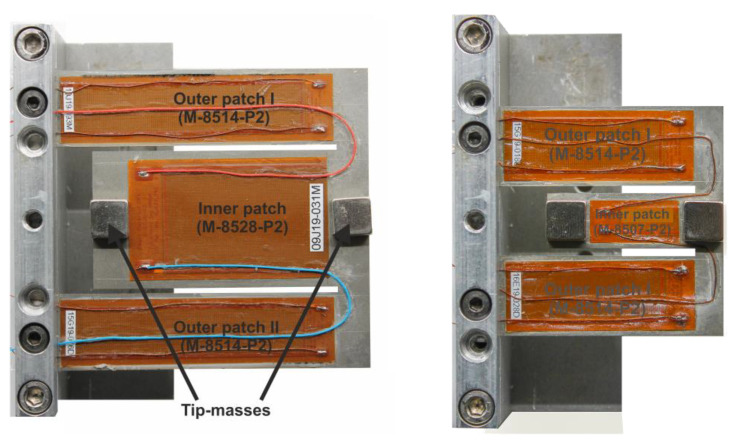
Optimized harvesters’ designs: design 1 (**left**), design 2 (**right**).

**Figure 13:**
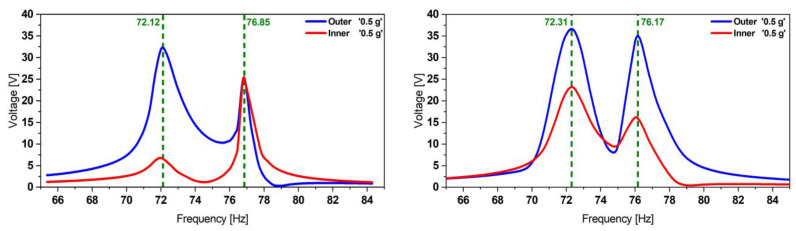
Optimized designs and their corresponding experimental transfer functions (**left**: design 1, **right**: design 2) exhibiting 3.2 times closer resonance frequencies and 2.9 times wider active bandwidth.

The optimization approach led to two design candidates (see Figure 12 and Figure 13) outperforming our reference design in terms of resonance frequency spacing and balanced power output. However, limited reproducibility in tip-masses and patch positioning as well steel thickness homogeneity led to a discrepancy in the expected and experimental performances of the harvester. This became most obvious in the case of design 2, with a steel thickness of 0.5 mm. 

Additionally, the tip-masses caused an obvious static deformation of the resonator, which led to dynamic properties differing from the undeformed design. Due to these inconsistencies, we proposed to proceed with the optimized design 1.

A full experimental characterization of the harvester, as well as a comparison with the predictions of the simulations, is given in detail in the following sections.

#### 3.2.1. Optimized Mechanical Resonator

**Figure 14:**
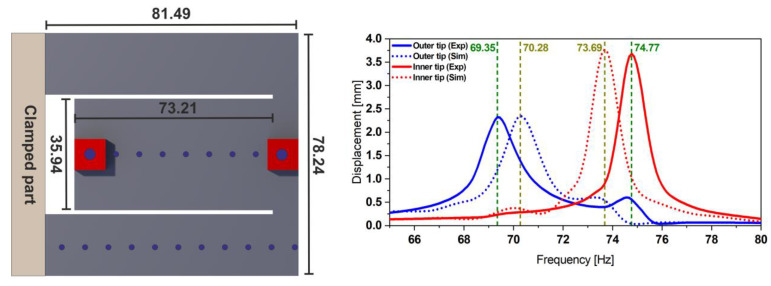
Geometry details of the coupled steel-based resonator (**left**); experimental displacement transfer function at 0.5 g (**right**), measured at each free end of the structure. The simulation results are also validated by comparing the mode shapes at the respective resonance frequencies.

Following the same procedure as in Section 2.2.1, we propose to experimentally characterize the mode shapes of the steel-based resonator and obtain the displacement transfer functions (see Figure 14). The results presented in Figure 14 show a good match between the experimental and the simulation data. A slight frequency shift and a discrepancy between the displacement amplitudes are caused by the uncertainty of the material properties and measurement equipment systematic errors as well as the mode-dependent damping ratio used in the FE model. 

We additionally observed similarities in the dynamics between the reference design and the proposed optimized design, reflected by the same mode shapes profile. The two fundamental modes indeed appear at two close frequencies (up to 3.2 times closer), enabling the broadening of the targeted harvesting operative frequency range. 

#### 3.2.2. Harvester Characterization and Power Estimation

**Figure 15:**
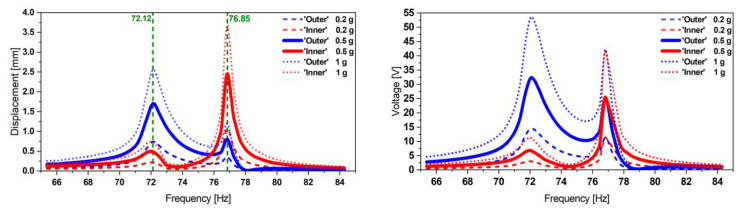
Experimental displacement (**left**) and corresponding voltage transfer functions (**right**) of the optimized design 1, appearing at two distinct but closer frequencies compared to the initial reference design. The tests were performed at various base acceleration levels.

We proceed with characterizing the proposed optimized design. We noticed a shift in terms of frequencies compared to the resonator resonance. We attribute this to the integration of the patches, which adds bending stiffness to the system. The displacement and voltage transfer functions at different excitation levels are depicted in Figure 15.

**Figure 16:**
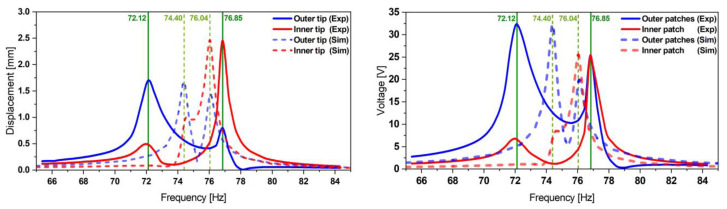
Experimental validation of the displacement (**left**) and the corresponding voltage (**right**) transfer functions obtained from the optimized design 1 FE model at an excitation of 0.5 g. It exhibits a good match in terms of amplitudes but a 2.9 times wider frequency-offset compared to the simulation expectations.

The experimental data were compared to the simulation results (see Figure 16). By matching the damping ratios, we could fit the simulated voltage amplitude to the experimental value of the optimized harvester with a remaining relative deviation of 0.5%. However, we additionally observed on three samples that the experimental frequency spacing was 2.9 times broader than the one expected in simulations. 

This discrepancy can be due to multiple reasons, such as the mode-dependent damping ratio used in simulations; the MFC geometry simplification in our FE model and where they have been considered as a complete piezoelectric sheet instead of real microfibers; and the manual gluing procedure used for integrating the piezoelectric patches, which makes controlling the glue layer thickness a challenging task.

In addition to these, there is the viscoelastic nature of the glue layer used for bonding the patches to the steel resonator, which was not reflected in the FE model, where we considered the bonding layer to be of a perfect bonding nature, as well as the fabrication tolerances. However, the mean relative frequency shift between the simulation and the experimental resonances is about 2.1%.

In order to estimate the power output of the system, we performed a load-matching procedure. The results presented in Table 4 show the power delivery of the optimized harvester design. It is also demonstrated that the design in question outperformed the reference design and could deliver 1.8 times higher power output. This is due to the higher induced deformation.

**Figure 17:**
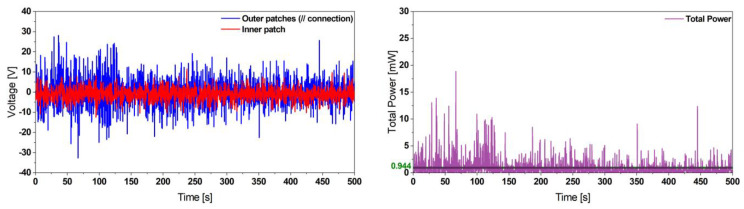
Experimental voltage (**left**) and instantaneous power output (**right**) results of the optimized harvester design 1 undergoing a white noise excitation providing a power density of 0.005 g^2^/Hz. It shows a higher power output compared to the initial reference harvester design.

Furthermore, we tested the performance of our optimized design under a under white noise excitation in the frequency range [50, 100] Hz and providing a power density of 0.005 g^2^/Hz. The voltage output is shown in Figure 17. The presented results demonstrate that our system is able to deliver a net power of 944 µW at the random excitation used for this test.

#### 3.2.3. Power Management

We employed the power management board 2151A described in detail in Section 2.2.3. The maximum power conversion efficiency was found to be 58%, as given in Table 5. The system was characterized at the 0.5 g excitation level.

#### 3.2.4. Summary

**Figure 18:**
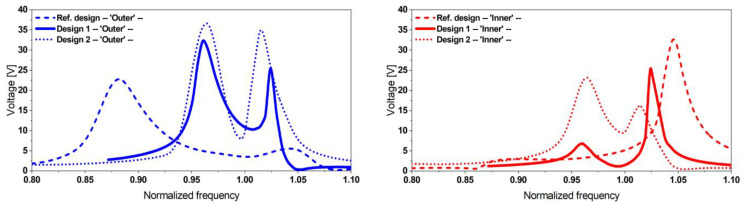
Comparison of the optimized and reference designs demonstrating that the optimized designs provide a 2.9 times wider harvesting bandwidth and 3.2 times closer resonances (see Table 6).

The data in Table 6 summarize the performances between the proposed optimized designs and the reference design. They clearly demonstrate that the optimized designs deliver higher power output. They also provide a wider active bandwidth, as demonstrated in Figure 18. The so-called ‘active bandwidth’ in our case represents the frequency range, where the harvester’s output voltage exceeds the minimum operating voltage of the power management board (5 V). For the sake of a fair comparison, we propose to normalize the frequency spacing to a central frequency *f*_0_ = 75 Hz.

Throughout this section, we thoroughly characterized an optimized version of our dual-frequency vibration-based energy harvester. It features a wider active harvesting bandwidth, with a resonance frequency spacing of 4.7 Hz. As stated above, the harvesting bandwidth is given by the frequency range in which the output voltage exceeds the minimum operating voltage of the power management board. The experimental characterization of the harvester demonstrated that the power delivery of the optimized design was 80% higher than the reference model. This means that we were able to develop a system which outperformed the initial design, although the power density was slightly lower (see Table 6), due to the larger size of the harvester. 

#### 3.2.5. Comparison between the Coupled and Simple Cantilever Array Harvester Designs

**Figure 19:**
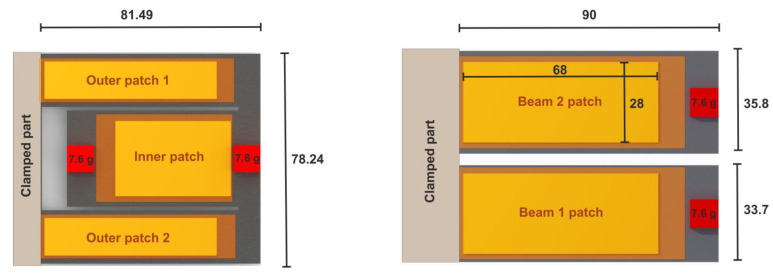
Geometry of the optimized harvester design 1 (**left**); two cantilever array harvester design of equal footprint to design 1 (**right**).

In order to demonstrate the applicability of our design and its optimization approach, we propose to compare the performances of our coupled resonator design with two individual cantilevers, as depicted in Figure 19. The total footprint of the two individual beams is equal to the optimized design.

**Figure 20:**
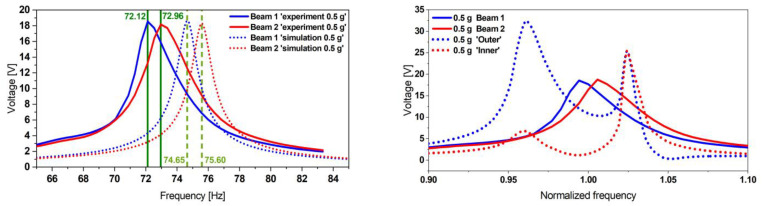
Experimental validation of the cantilever array-based harvester FE model (**left**) and the comparison of the proposed optimized harvester design 1 with its equivalent cantilever array-based harvester (**right**) revealing that coupled design outperforms its equivalent by delivering two times higher power output (see Table 7) and five times wider active bandwidth.

The experimental results presented in Figure 20 show a good match with the simulation results, with matched damping ratio values in terms of voltage level. However, a maximum mismatch in the frequencies of 3.6% was observed. We additionally noticed an increasing frequency shift and nonlinear effect on the experimental transfer function shape while increasing the base excitation levels, which is not reflected in the simulation data.

The comparison of the voltage and the power delivery of the two designs revealed that the folded beam design outperforms the two individual cantilever-based design by delivering two times higher power output, as represented in Table 7. This is due to the higher deformation on the coupled resonator compared to the two decoupled cantilever-based resonator. Additionally, the coupled resonator yielded higher overall displacements, which resulted in higher induced deformation, when compared to the simple cantilever-based resonator.

However, the simple cantilever array provides geometric simplicity and exhibits the ability to adjust the design parameters to yield two close frequencies. Throughout this work, we demonstrated the feasibility of achieving coupled designs which outperform their equivalent decoupled designs. The presented FE models are capable of predicting the dynamic behavior of both designs. 

## 4. Conclusions and Outlook

Throughout this work, we introduced a dual-frequency vibration-based energy harvester based on coupled resonators. The dual-frequency feature was investigated, and we demonstrated that the resonator magnified the excitation amplitude at two close fundamental resonance frequencies, enabling simultaneous energy harvesting from two vibration frequencies. The experimental data showed a good match with the simulation expectations. Furthermore, it was demonstrated that the harvester and a power management module were capable of delivering 500 µW, which is a typical value for low-power applications.

An optimization strategy resulted in optimized versions of the proposed harvester design. Their experimental characterization revealed that the resonance frequencies were 3.2 times closer compared to the reference model. The designs also exhibited higher power output as well as wider harvesting bandwidth. The experimental investigations validated the FE model, such that the experimental data showed a good match with the simulation results. The experimental characterization of the optimized designs showed a wider harvesting bandwidth and resonances appearing at a spacing of 4 Hz instead of 2 Hz, as expected in simulations. This was due to the fabrication tolerances and the piezoelectric material property simplifications considered in the FE model. 

Furthermore, the proposed optimized designs were compared to their equivalent decoupled cantilevers design. The comparison revealed that the proposed designs outperformed their standard harvester competitors by a factor of two in terms of power output as well as active bandwidth. However, the decoupled cantilevers can be designed in a simple way. 

The present work adds to numerous previous research efforts dedicated to investigating the use of coupled resonator designs for harvesting purposes. The proposed designs deliver higher power outputs over wider harvesting bandwidths compared to previously proposed coupled resonator-based energy harvesters, such as in [19]. This was achieved by bringing the two fundamental resonance frequencies as close as possible and avoiding the mode steering phenomenon.

Finally, the optimization approach provided us with candidate designs for a folded harvester with better performances compared to the initial design. However, we observed an uncertainty in the expected power delivery and frequency range. Therefore, in future works, we propose to improve the quality of our optimization strategy by including a better damping estimation approach. Furthermore, an optimization of the assembly and fabrication process is planned. The developed harvesters will be tested in real-time applications, e.g., for powering a battery-free sensor node.

## Figures and Tables

**Table 1:** Approximated material properties of the active area of the Macro Fiber Composite^TM^ patches.

Material Property	Value
Mass density (kg/m^3^)	5440
Tensile modulus, *E*_1_ (rod direction) (GPa)	30.34
Tensile modulus, *E*_1_ (electrode direction) (GPa)	15.86
Poisson’s ratio, *v*_12_	0.31
Poisson’s ratio, *v*_21_	0.16
Shear modulus, *G*_12_ (GPa)	5.515
*d*_33_ (rod direction) (pC/N)	400
*d*_31_ (electrode direction) (pC/N)	−170

**Table 2:** Harvester reference design characterization at 0.5 g excitation level, using the 2151A power management board. (***f:*** frequency, ***V_in_***: voltage input, ***I_in_***: current input, ***P_in_***: power input, ***R***: resistive load, ***V_out_***: voltage output, ***I_out_***: current output, ***P_out_***: power output).

Patch	*f* [Hz]	*V_in_* [V]	*I_in_* [μA]	*P_in_* [mW]	*R* [kΩ]	*V_out_* [V]	*I_out_* [μA]	*P_out_* [mW]	Efficiency [%]
Outer	65.03	10.12	254.0	2.57	3.8	2.500	638.3	1.590	59.1
Inner		1.38	n.a.	n.a.	n.a.	0	0	0	n.a.
Outer	77.78	3.889	97.2	0.378	32.0	2.5	77.49	0.193	51.1
Inner		12.51	323.4	4.046	2.4	2.5	996.3	2.482	61.4

**Table 3:** Reference and optimized harvesters’ geometry details (***l_1_*:** outer beam length, ***l_2_:*** inner beam length, ***w_1_*:** outer beam width, ***w_2_:*** inner beam width, ***h:*** resonator thickness, ***l_p1_*:** outer patch length, ***l_p2_*:** inner patch length, ***w_p1_:*** outer patch width, ***w_p2_*:** inner patch width, ***h_p_*:** piezo-patch thickness).

Parameters [mm]	Reference Design	Design 1	Design 2
***l_1_***	80.0	81.49	65.41
***l_2_***	60.0	73.21	36.70
***w_1_***	10.0	19.88	56.84
***w_2_***	18.0	35.94	13.62
***h***	01.0	1.000	0.500
***l_p1_***	60.0	64.21	39.45
***l_p2_***	48.0	43.10	14.65
***w_p1_***	07.0	14.00	14.00
***w_p2_***	14.0	28.00	07.00
***h_p_***	0.18	0.18	0.18

**Table 4:** Optimized harvester design 1 experimental load-matching and instantaneous power characterization at 0.5 g excitation level.

Patch	*f* [Hz]	*V*_out_ [V]	*R* [kΩ]	*I*_out_ [mA]	*P*_out_ [mW]	*P*_total_ [mW]
Outer	72.12	14.32	15.0	0.955	27.34	28.57
Inner		2.712	15.0	0.209	1.226	
Outer	76.85	7.446	15.0	0.496	7.392	13.63
Inner		6.117	15.0	0.510	6.236	

**Table 5:** Optimized harvester design 1 characterization at 0.5 g excitation level using the 2151A power management circuit.

Patch	*f* [Hz]	*V*_in_ [V]	*I*_in_ [mA]	*P*_in_ [mW]	*R* [kΩ]	*V*_out_ [V]	*I*_out_ [mA]	*P*_out_ [mW]	Efficiency [%]
Outer	72.12	14.10	1.066	15.03	0.328	2.500	3.280	8.200	54.56
Inner		3.633	0.157	0.570	2.100	2.500	0.118	0.295	51.72
Outer	76.85	8.112	0.450	3.650	3.000	2.500	0.804	2.010	55.06
Inner		8.015	0.394	3.158	3.300	2.500	0.733	1.832	58.03

**Table 6:** Optimized designs’ maximum raw power characterization and comparison with the initial reference harvester design.

Design	*f*_1_ [Hz]	*f*_2_ [Hz]	∆*f/f*_0_ [%]	*P* [mW]	Footprint [cm^2^]	Power Density [µW/cm^2^]
Ref. design	66.16	78.42	16.34	15.74	32.0	491.9
Opt. design 1	72.12	76.85	6.308	28.50	62.13	458.7
Opt. design 2	72.31	76.17	5.147	28.75	37.18	773.3

**Table 7:** Experimental performance comparison between the coupled resonator-based optimized design 1 and its equivalent simple cantilever array-based harvester.

Design	*f*_1_ [Hz]	*f*_2_ [Hz]	∆*f/f*_0_ [%]	*P* [mW]
Design 1	72.12	76.85	6.308	28.50
Cantilevers	72.12	72.96	1.159	13.75
